# The Dynamics of Natural *Plasmodium falciparum* Infections

**DOI:** 10.1371/journal.pone.0045542

**Published:** 2012-09-18

**Authors:** Ingrid Felger, Martin Maire, Michael T. Bretscher, Nicole Falk, André Tiaden, Wilson Sama, Hans-Peter Beck, Seth Owusu-Agyei, Thomas A. Smith

**Affiliations:** 1 Swiss Tropical and Public Health Institute, Basel, Switzerland; 2 University of Basel, Basel, Switzerland; 3 Kintampo Health Research Centre, Kintampo, Ghana; London School of Hygiene and Tropical Medicine, United Kingdom

## Abstract

**Background:**

Natural immunity to *Plasmodium falciparum* has been widely studied, but its effects on parasite dynamics are poorly understood. Acquisition and clearance rates of untreated infections are key elements of the dynamics of malaria, but estimating these parameters is challenging because of frequent super-infection and imperfect detectability of parasites. Consequently, information on effects of host immune status or age on infection dynamics is fragmentary.

**Methods:**

An age-stratified cohort of 347 individuals from Northern Ghana was sampled six times at 2 month intervals. High-throughput capillary electrophoresis was used to genotype the *msp-2* locus of all *P. falciparum* infections detected by PCR. Force of infection (FOI) and duration were estimated for each age group using an immigration-death model that allows for imperfect detection of circulating parasites.

**Results:**

Allowing for imperfect detection substantially increased estimates of FOI and duration. Effects of naturally acquired immunity on the FOI and duration would be reflected in age dependence in these indices, but in our cohort data FOI tended to increase with age in children. Persistence of individual parasite clones was characteristic of all age-groups. Duration peaked in 5–9 year old children (average duration 319 days, 95% confidence interval 318;320).

**Conclusions:**

The main age-dependence is on parasite densities, with only small age-variations in the FOI and persistence of infections. This supports the hypothesis that acquired immunity controls transmission mainly by limiting blood-stage parasite densities rather than changing rates of acquisition or clearance of infections.

## Introduction

In highly endemic areas, the transmission of *Plasmodium falciparum* malaria is in a state of endemic equilibrium, implying that each infection on average replaces itself with a single infection in the next parasite generation. This is despite basic reproduction numbers that can be in the thousands. This control of transmission is achieved by naturally acquired immunity, the effects of which must therefore be considered in models for *P. falciparum* dynamics in endemic settings [1]. Different malariologists make different assumptions, often implicitly, about what are the effects against different parasite stages of naturally acquired immunity and how this translates into control of transmission. These assumptions, about the persistence of infections, infectiousness, and the effects of exposure to the parasite on the risk of subsequent super-infection, are critical components of models of parasite dynamics and of the impact of preventive interventions.

The clearest manifestations of acquired immunity are on asexual parasite densities and incidence of clinical disease. Some models of malaria dynamics, for instance [2], [3], invoke major effects of natural immunity on the rate at which infections are acquired (pre-erythrocytic immunity) or cleared. There have been very few empirical comparisons of acquisition and duration of natural infection between groups with different immune status or histories of exposure.

The lack of a general immunological correlate of protection, and the challenge of measuring histories of exposure at the individual level means that the most practicable exposure measure is often the host age. In endemic areas, levels of anti-malarial antibodies and other effectors generally increase with age [4]–[6], (though specific responses vary in persistence and age profiles), making age a rough proxy of immune status.

The acquisition of infection can in principle be easily measured in relation to host age by clearing infections and observing time to re-infection, but there is only one available dataset of this type for the whole age-range of hosts. This is from Garki study [7] and suggests that the force of infection (FOI; defined as the average number of new infections acquired by a host per unit time) increases with host age over the first few years of life [8], consistent with data indicating that larger people are bitten by more mosquitoes [9], [10]. These data also suggest that FOI is reduced in older hosts, presumably due to acquired immunity.

The duration of infection was studied in the mid-20th century by interrupting transmission and examining the time taken for infections to clear [11]. However time to clearance depends on the number of co-infecting parasite clones (multiplicity of infection, MOI), which could not be determined before the advent of genotyping. The duration of a clonal infection is difficult to determine in field samples from endemic areas because of frequent super-infections, and in longitudinal studies with short term sampling (daily or weekly) of asymptomatic individuals, individual parasite clones frequently appear to be lost only to reappear again [12]. Part of the explanation is sequestration of cyto-adhering late-stage parasites, so that well-synchronised parasite clones can escape detection. A parasite clone can also be undetected because its density falls below the detection limit, even that of nested PCR (nPCR). Temporary absence from the peripheral blood must be distinguished from parasite clearance, and estimates of both FOI and duration that fail to account for imperfect detection (e.g. [13]–[15]) are strongly biased downwards.

Standard molecular methods detect at most about half of all clones present in the host [16]–[18], mainly because there is often no template in the blood volume analyzed. Increasing the blood volume for DNA extraction augments detectability, but this will never reach 100% because of sequestration of some clones. More clones can be collected by repeated sampling at short intervals but even collection of four samples over eight days does not guarantee detection of all the clones in a host [18]. The challenge of imperfect detection of DNA thus needs to be addressed by statistical methods that simultaneously consider both the parasite dynamics and detection process [16], [18]–[20]. Such statistical methods recognize that failure to detect a particular genotype at a date between two positive samples is consistent with the presence of the parasite at a density below the detection limit.

Until recently there were no datasets covering the whole range of ages, from which patterns of FOI and duration with age could be estimated using these approaches. We therefore carried out a cohort study of an age-stratified sample of 347 people in a highly endemic setting in Ghana. Blood samples were collected in two-monthly intervals over one full year. The genotyping data obtained in a pilot sub-sample of 69 of these individuals was used for analysis of FOI and duration by age [20], [21], [21,22], but was too sparse to allow us to fit separate models to different age groups and to analyze non-monotonic patterns of age dependence.

We also recently established a high though-put genotyping technique based on PCR fragment sizing by capillary electrophoresis (CE) facilitating longitudinal tracking of multiple infections using an automatic readout [17]. We used this to expand the pilot analyses of parasite typing and dynamics to include data from all 347 individuals. This has enabled us to compute age-specific estimates of FOI, infection duration and detectability, and hence to test the conjecture that both FOI and clearance rates vary non-monotonically with age in endemic areas.

**Figure 1:**
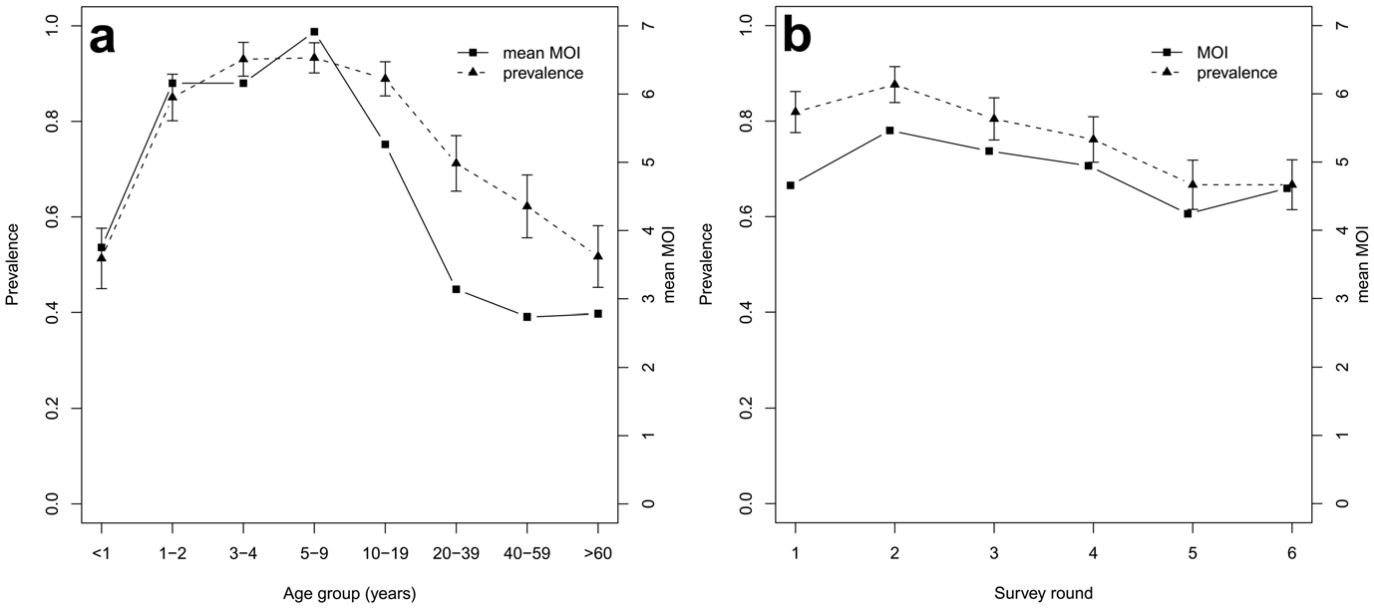
Mean multiplicity of infection (MOI) and prevalence by PCR. (**a**) by age and (**b**) by survey. Mean multiplicity is calculated from PCR positive samples only. Error bars correspond to approximate 95% confidence intervals.

## Materials and Methods

### Study Site and Field Methodology

The study was carried out in Kassena-Nankana District (KND), northern Ghana where *P. falciparum* transmission levels (Entomological Inoculation Rates) of over 300 infective bites per person per annum have been documented. The main vectors are *Anopheles gambiae* s.l. (both *An. gambiae* s.s. and *An. arabiensis* and *An. funestus*. *P. falciparum* infection in KND shows seasonal peaks and troughs in prevalence and clinical malaria incidence, with an incidence density of infection of 5 infections per person-year in the dry season to as high as over 7 cases per person-year in the wet season [22]–[23]. Sampling methods were described previously [22]. Blood samples were collected on DNA ISOCodeTM Stix (Schleicher & Schuell) at intervals of two months, starting in 2000, resulting in a total of 6 samples per participant (R1-R2-R3-R4-R5-R6). Participants who were sick at a survey date were referred to routine health services. No antimalarial treatments were administered by the research team. Informed consent was obtained from participants by signature or thumbprint in the presence of a witness. Ethical clearance for this study was obtained from the Ghana Health Service Ethics Committee.

**Figure 2:**
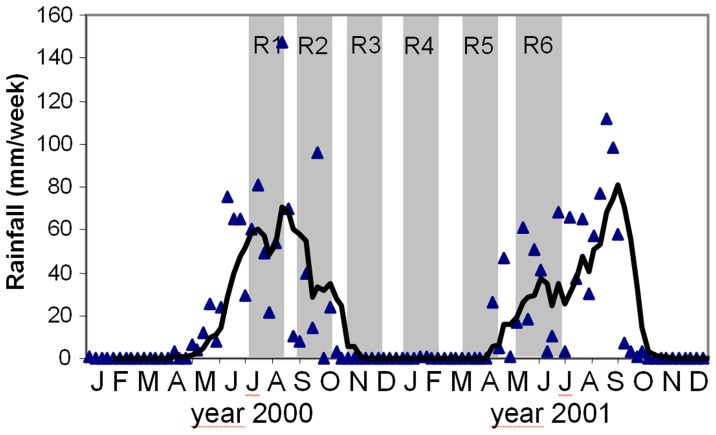
Sampling intervals and rainfall in Navrongo during the study period. Blue triangles represent weekly rainfall, the black line shows the 5 weeks moving average of rainfall. Grey bars represent the six sampling periods.

### Laboratory Methods

DNA extraction and PCR were described previously [17], [24]. The CE-based genotyping technique used differentially labeled fluorescent dyes for the two allelic families of the polymorphic marker gene *msp-2*. This typing technique had been validated with a subset of our samples [17]. An in-house generated program was used to classify peaks sized by GeneMapper® software. Post-processing procedures were described previously [17].

**Figure 3:**
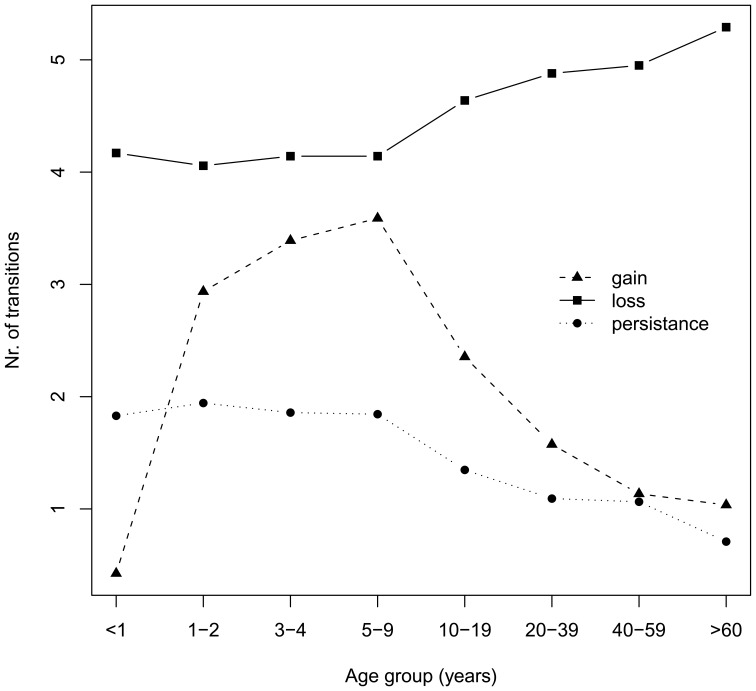
Transition types observed between two consecutive survey rounds by age group. Transitions are gains or losses of parasite clones.

### Data Analysis

The genotyping data were first analyzed by calculating observed frequencies of apparent gain, loss and persistence of infecting clones. An infection present in the survey at time t, but not detected in the subsequent survey t+1 was considered a “loss” (+ −), whereas a “gain” (− +) was counted when an infection was observed in round t but not in the previous round t-1. Where infections were observed in consecutive surveys, this was recorded as “persistence”.

This exploratory analysis did not allow for imperfect detection. Two different methods were used to simultaneously estimate detectability and duration of infection allowing for undetected infections. Comparing the results of the two analytical approaches makes it possible to distinguish real effects from any artifacts specific to one or other statistical model.

**Table 1:** Characteristics of Cohort Studied.

No. individuals enrolled	347
No. of samples collected	1902
No. of samples analyzed by PCR	1803
No. of individuals with a complete set of 6 samples	269
No. of these individuals with at least one PCR positive sample	240
No. of these individuals with more than one parasite-positive blood sample.	216
% Female	53
*P. falciparum* positivity by microscopy	(proportion)
<1yr	0.453
1–2 yrs	0.797
3–4 yrs	0.786
5–9 yrs	0.739
10–19 yr	0.624
20–39 yrs	0.469
40–59 yrs	0.368
>60 yrs	0.328
overall	0.575
*P. falciparum* positivity by PCR, all ages	0.729
Geometric mean parasite density by age group (95% CL)	
<1yr	770.6(500.1, 1187.6)
1–2 yrs	1053.6(766.7, 1447.9)
3–4 yrs	1064.5(805.3, 1407.2)
5–9 yrs	420.5(344.5,513.2)
10–19 yr	291.0(233.4, 362.9)
20–39 yrs	111.4(84.5, 146.9)
40–59 yrs	119.8(84.2, 170.4)
>60 yrs	116.8(89.9, 151.8)

**Table 2:** Frequencies of different observed patterns of appearance and re-appearance of parasite genotypes.

Survey number						Frequency
1	2	3	4	5	6	
−	−	−	−	−	+	271
−	−	−	−	+	−	257
−	−	−	−	+	+	59
−	−	−	+	−	−	390
−	−	−	+	−	+	41
−	−	−	+	+	−	46
−	−	−	+	+	+	25
−	−	+	−	−	−	445
−	−	+	−	−	+	29
−	−	+	−	+	−	25
−	−	+	−	+	+	19
−	−	+	+	−	−	73
−	−	+	+	−	+	23
−	−	+	+	+	−	16
−	−	+	+	+	+	18
−	+	−	−	−	−	577
−	+	−	−	−	+	34
−	+	−	−	+	−	28
−	+	−	−	+	+	12
−	+	−	+	−	−	49
−	+	−	+	−	+	9
−	+	−	+	+	−	8
−	+	−	+	+	+	11
−	+	+	−	−	−	71
−	+	+	−	−	+	12
−	+	+	−	+	−	13
−	+	+	−	+	+	10
−	+	+	+	−	−	29
−	+	+	+	−	+	11
−	+	+	+	+	−	8
−	+	+	+	+	+	15
+	−	−	−	−	−	454
+	−	−	−	−	+	23
+	−	−	−	+	−	18
+	−	−	−	+	+	4
+	−	−	+	−	−	25
+	−	−	+	−	+	6
+	−	−	+	+	−	8
+	−	−	+	+	+	4
+	−	+	−	−	−	49
+	−	+	−	−	+	9
+	−	+	−	+	−	5
+	−	+	−	+	+	3
+	−	+	+	−	−	15
+	−	+	+	−	+	8
+	−	+	+	+	−	4
+	−	+	+	+	+	6
+	+	−	−	−	−	75
+	+	−	−	−	+	9
+	+	−	−	+	−	11
+	+	−	−	+	+	8
+	+	−	+	−	−	10
+	+	−	+	−	+	6
+	+	−	+	+	−	7
+	+	−	+	+	+	5
+	+	+	−	−	−	20
+	+	+	−	−	+	6
+	+	+	−	+	−	6
+	+	+	−	+	+	3
+	+	+	+	−	−	8
+	+	+	+	−	+	14
+	+	+	+	+	−	4
+	+	+	+	+	+	18

The frequencies of the patterns are summed over all hosts and genotypes. + indicates detection of the genotype by CE; - indicates that it was not detected at that survey. Each of the 63 possible patterns occurred at least 3 times.

**Figure 4:**
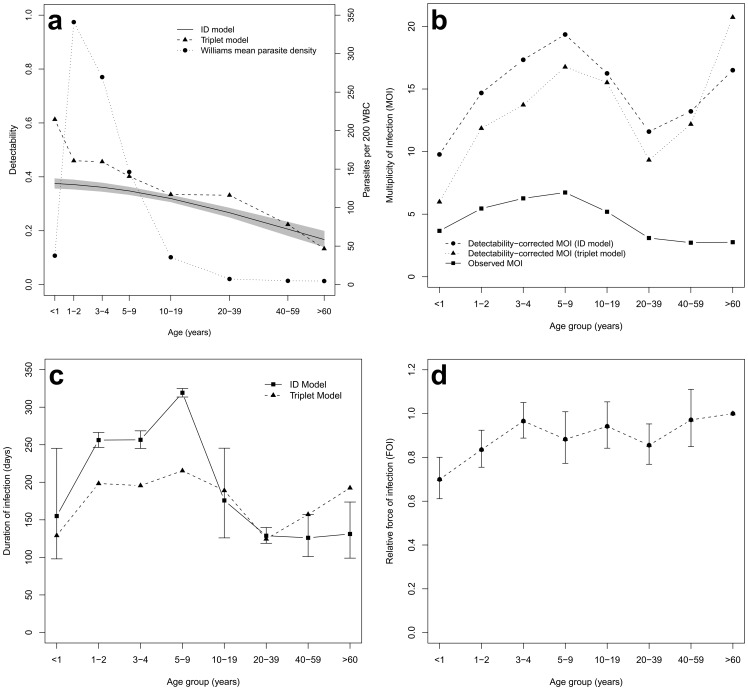
Age dependence of parameter estimates. **a.** Estimated detectability by age (Model A and triplet model), and Williams mean parasite densities by age group assessed by microscopy. The grey area is the 95% confidence envelope for the detectability. (The Williams mean of *N* observed densities *y* is calculated as 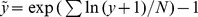). **b.** Observed mean multiplicity of infection (MOI) by age group in northern Ghana and estimated MOI allowing for imperfect detection. MOI was determined from PCR positive blood samples only. **c.** Estimated average duration of an infection by age group (Model A). Error bars are approximate 95% confidence intervals. **d.** Estimated force of infection by age group (Model B). Estimates are rates expressed relative to that in the oldest age group. Error bars are approximate 95% confidence intervals.

For the “triplet” approach the data were arranged as records of presence or absence of individual parasite genotypes in sequences of up to three successive samples from the same individual. A sliding window of three consecutive samples was used so that each sample could appear at any position in the triplet. Four types of patterns were counted (++x; +−+; +−; +−?; where + represents positive, – negative, ? missing, and x can be any of the three). The expected relative frequencies of these patterns were calculated assuming parasite persistence to be a homogeneous first order Markov process, and parasite detection to be described by a constant detectability parameter [16]. Bayesian estimates of both detectability and clearance rates were made assuming a multinomial distribution for the frequencies of the different kinds of triplets. Separate estimates were made for each age group using the software Winbugs version 1.4 [25].

**Table 3:** Parameter Estimates of Statistical Models.

Parameter		Unit	Value	
			ID model	Triplet model
λ_1_	Force of Infection Aug/Sep	per yr	44.8 (CI 44.7, 44.9)	n.a.
λ_2_	Force of Infection Oct/Nov	per yr	19.2 (CI 19.1, 19.3)	n.a.
λ_3_	Force of Infection Dec/Jan	per yr	18 (CI 17.8, 18.2)	n.a.
λ_4_	Force of Infection Feb/Mar	per yr	7.5 (CI 7.4, 7.6)	n.a.
λ_5_	Force of Infection Apr/May	per yr	12.3 (CI 12.2, 12.3)	n.a.
λ_6_	Force of Infection Jun/Jul	per yr	39.6 (CI 39.3, 39.8)	n.a.
1/µ_1_	Duration of infection (<1 yr)	days	155 (CI 128, 183)	129 (CI 108, 160)
1/µ_2_	Duration of infection (1–2 yrs)	days	256 (CI 254, 259)	198 (CI 143, 322)
1/µ_3_	Duration of infection (3–4 yrs)	days	257 (CI 254, 259)	196 (CI 162, 247)
1/µ_4_	Duration of infection (5–9 yrs)	days	319 (CI 318, 320)	216 (CI 169, 297)
1/µ_5_	Duration of infection (10–19 yrs)	days	176 (CI 156, 196)	189 (CI 143, 279)
1/µ_6_	Duration of infection (20–39 yrs)	days	129 (CI 124, 134)	124 (CI 93, 186)
1/µ_7_	Duration of infection (40–59 yrs)	days	126 (CI 113, 139)	158 (CI 95, 472)
1/µ_8_	Duration of infection (>60 yrs)	days	131 (CI 114, 148)	190 (CI 97, 5,924)
s_0_	Logit detectability	at 20 yrs	−0.84 (CI −0.91, −0.78)	n.a.
s_1_	Change in logit detectability	per 10 yrs	−0.17 (CI −0.21, −0.13)	n.a.
q_1_	Detectability (<1 yr)	percent	n.a.	61 (CI 48, 74)
q_2_	Detectability (1–2 yrs)	percent	n.a.	46 (CI 34, 58)
q_3_	Detectability (3–4 yrs)	percent	n.a.	46 (CI 39, 52)
q_4_	Detectability (5–9 yrs)	percent	n.a.	40 (CI 34, 47)
q_5_	Detectability (10–19 yrs)	percent	n.a.	33 (CI 26, 41)
q_6_	Detectability (20–39 yrs)	percent	n.a.	33 (CI 19, 48)
q_7_	Detectability (40–59 yrs)	percent	n.a.	22 (CI 09, 35)
q_8_	Detectability (>60 yrs)	percent	n.a.	13 (CI 03, 23)

ID, immigration-death, model A; n.a., not applicable, i.e. the estimation method did not provide these estimates. The force of infection estimates include a scale factor to allow for the exclusion of uninformative individuals (those with no detected infections) from the analysis.

The second approach, an immigration-death (ID) model, considered patterns consisting of sequences of all six observations allowing for seasonal variation in the force of infection and imperfect detection of an infection at some of the time points [20], [21]. Detectability, FOI and duration of an infection were simultaneously estimated by maximum likelihood fit to the frequency distributions of the patterns of six observations for each observed infection. *n(a),* the expected number of distinct genotypes (or the expected true MOI) within an individual of age *a* is given by the differential equation [20], [21]:
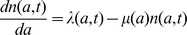
where 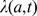 is the infection rate and 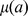
is the clearance rate at age *a*. We assumed that this process has continued since birth, and that 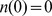, leading to dynamics corresponding to the McKendrick-von Foerster population model [26].

In previous analyses [21], for which only the pilot dataset was available, we considered 12 such ID models in which the clearance rate was either age independent or a monotonic function of age. FOI was either constant, varied seasonally, or varied monotonically with age. The best fitting model had seasonally varying FOI and constant clearance rate. With the much larger dataset now available we extended the analysis to consider non-monotonic relationships with age (binned into 8 categories) in both 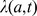 and 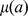.

In the previous best fitting model [21], the clearance rate, µ, was an exponential function of age, while in the current analyses we fitted separate clearance rates *µ_a_*, for each age group *a*, where the *a* denotes the age on acquisition of the infection.

We fitted the two distinct ID models with different parameterizations of the infection process:
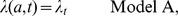
corresponding to the previous(4) best fitting model, where 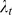 is a season-specific FOI, or:



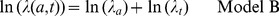
where 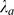 is the age-specific FOI for age group *a* (relative to the oldest age group). This allows for more flexibility in representing the dependence of FOI on age of the host and allowed us to test whether FOI peaks at intermediate ages.

Following the previous analyses [21], we parameterized the detectability *s* in both Model A and Model B as a logistic function of age, i.e.:
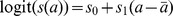
where 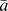 is the mean age and *s_0_* and *s_1_* are parameters to be estimated. We compared the fit of models A and B, and with the fit of the previous best fitting model, using the Akaike Information Criterion (AIC) [21]. Further details of this statistical approach were described previously [20], [21]. The present study is the first analysis that tests whether there is a maximum in the duration at some intermediate age when allowing for varying detectability.

A JAVA program implementing the ID models is available at: https://bitbucket.org/mthbretscher/amalid.

## Results

### Data Description

From the 347 individuals enrolled at baseline, 1902 blood samples were collected during the one year follow-up in two-monthly intervals (Supplementary Table S1). All 1386 PCR positive blood samples were genotyped by our highly accurate technique. This amounted to a total of 6386 detected PCR fragments. The GeneMapper® analysis distinguished 103 different msp2 genotypes (Supplementary Table S1). 28 belonged to the FC27 allelic family and 75 were of 3D7-type. FC27-type alleles generally reached higher allelic frequencies than 3D7 genotypes. The most frequent genotype represented 10.2% of all fragments detected (651/6386) and was of FC27 type. The most frequent 3D7 genotype represented 3.6% of all fragments.

In the overall data set *P. falciparum* prevalence was 57.5% by microscopy and 73% by PCR. The age distribution of PCR positivity showed a peak in the 5–9 year old children with 93% of these children being parasite positive (Figure 1a). Mean multiplicity of infection (MOI), measured in PCR positive blood samples, also peaked in the age group of 5–9 years. Both prevalence and MOI were lowest in the 60+ age group. The highest prevalence by PCR (88%) and highest MOI (a mean of 5.5 clones per individual) was observed in Round 2 (Figure 1b). These peaks coincided with the rainy season as sampling for Round 2 started just after the rainfall had reached a maximum (Figure 2). Prevalence was lowest (67%) in Round 6, which marked the beginning of the next rainy season.

The apparent dynamics of *P. falciparum* infections can be described by the observed rates of clone appearance and disappearance (Figure 3). This analysis ignores effects of imperfect detectability. Rates of gain were higher in children than in adults, with a peak in children aged 5–9 years. The observed rate of clone disappearance showed an opposite trend. More clones were lost between two surveys in older individuals than in young children. The apparent persistence of clones thus decreased with age.

### Detectability

The data set used in the present analysis to estimate detectability and duration of infection from longitudinal genotyping data contained 240 individuals with complete records for each round and at least one parasite positive sample (Table 1). A total of 3485 different infections (corresponding to different host/msp-2 genotype combinations) were detected in these 240 hosts, with the frequencies of the different patterns of appearance and reappearance in the six surveys given in Table 2. Both statistical methods for estimating detectability found that even highly sensitive nPCR combined with precise CE- GeneMapper® based fragment sizing detected less than half of the clones present at a given time in an individual (Figure 4a, Table 3). Both statistical methods also agreed that in younger age groups <10 years, detectability was higher than in older ages, reaching a minimum of 17% in the oldest individuals. This age-dependent decline of detectability is also in line with microscopic parasite density decreasing with age (Figure 4a). Our implementation of the immigration-death model constrained the relationship of detectability with age to be monotonic, leading to a decrease in detectability with age. Overall mean detectability in all six rounds was estimated to be 30% by the ID model.

### Multiplicity of Infection

Estimates of the age profile of MOI also need to be corrected for the effects of imperfect detection of parasite clones. Figure 4b shows the observed versus the true MOI corrected for imperfect detection by both models. The age pattern of MOI, adjusted for detectability, was similar, whichever model was used to estimate detectability. Estimated MOI in infants was lowest at roughly 10 infecting clones per child. There was a peak in 5–9 year old children with about 19 concurrent infections per child. All ages >20 years showed a lower MOI when adjusted for detectability by the ID model (model A). Overall, correction with the immigration death model provided higher estimates of MOI and gave estimates of overall mean MOI reaching a maximum of 18 at the end of the wet season and a minimum of 14 at the end of the dry season, assuming an average detectability of 0.3.

MOI observed in our study population was thus high, though when age-adjusted it was lower than that in other studies in Ghana [27]. Even in high transmission areas MOI is generally low in the youngest children, and shows a peak at about the same age as the prevalence assessed by microscopy [22], [28], [29]. In lower transmission areas (including another area in Ghana [30]), MOI is both lower [31], [32] and less age-dependent [33]. This is consistent with the observed patterns of parasite densities by age (Figure 4a) which clearly imply that there is much more PCR template per clone in young children than in adults.

### Duration of Infection

The duration of infection was estimated by both modeling approaches (Figure 4c, Table 3). The “triplet” model gave estimates of duration much longer than those based on direct observations in blood samples, averaging 168 days (95% CI 144, 202) over the whole age range. Even longer estimates of mean duration (194 days, 95% CI 191, 196) were obtained using the ID approach. The ID approach makes more efficient use of the data and adjusts for seasonality in the infection process, and hence this estimate is to be preferred. Both models could also consider each age group separately and thus allow for non-monotonic age effects, and both indicated a peak in duration of infection in children 5–9 years of age (Figure 4c). Infection duration for infants and older ages were shorter. For the youngest age groups, short durations may partly reflect effects of antimalaria treatment, mainly affecting <2 years old children. Treatment was infrequent but could not be formally incorporated into the models because of absence of comprehensive individual-level data.

The fit of ID model A, which allowed for non-monotonicity in duration, was much better (AIC = 8005.1) than that of the previously best fitting ID model (model 6 in the analyses of the pilot dataset [21]), which gave an AIC value of 8127.4 when fitted to the full dataset [34].

### Force of Infection

Estimates of FOI by age group were available only from Model B, where FOI increased somewhat with age (Figure 4d). This contrasts with the earlier analyses, not allowing non-monotonicity in durations, which suggested that FOI might decrease slightly with age [21]. The AIC for Model B was 8012.7, higher than that of model A, indicating no statistically significant improvement in fit compared with the model that treated FOI as age-independent.

### Discussion

Age patterns of FOI, detectibility and duration of infection in *P. falciparum* result from the interplay of infection and clearance processes, but their analysis is complicated by imperfect detection of parasites. Our study brings together laboratory and statistical methods able to assess these and applies these to a large field study of a representative sample of people in an endemic setting. CE for sizing of PCR fragments enables accrual of much larger and more accurate longitudinal datasets than was possible with traditional side-by-side runs of PCR products or RFLP fragments on gels. This increases our confidence that effects of parasite clearance can be separated statistically from those of imperfect detection, allowing us to make much more precise estimates of the age-dependence in these parameters, in particular allowing us to test non-monotonic age dependency.

Analyses of longitudinal typing studies can be confused by the complex patterns of intermittent appearance of individual clones, which have sometimes been interpreted as rapid turnover [12]. Our studies strongly suggest that this is the result of imperfect detection. Both indirect evidence and common sense) suggests that this is likely to be related to fluctuations in density of persisting infections, which often fall below the detection limit of nPCR, rather than reinfection with an indistinguishable clone. The low frequencies of each individual genotype (Supplementary Table S1) imply that super-infections can usually be distinguished from other infections in the same host.

A key result of this analysis is that, once age-dependence in detectability is allowed for, FOI tends to increase, though not statistically signficantly, over the whole age range. This is in agreement with re-infection studies carried out in Navrongo over a decade ago. These studies found similar FOI in adults [35], and in young children [36], albeit during different time periods. This pattern is discordant with the data from the Garki project, in which FOI increased with age over the first decade of life but was lower in adults than in children. These appear to be the only empirical studies that include adults, for how this important parameter varies with age in endemic settings.

Estimates of the age-dependence of duration are also strongly modified by allowing for age-dependence in detectability. When this is ignored, parasite persistence appears to decrease strongly with age, corresponding to the idea that stronger immunity in older individuals clears infections. In a study in Papua New Guinea that did not consider detectability a median duration of clonal infections of >60 days was estimated in 4 year olds, but a median of only 15 days was estimated for children of 5–14 years [15].

By allowing for imperfect detection, the present study found a very different pattern, with the duration of infection somewhat shorter in infants than in older children (Figure 4c). This is in agreement with our earlier findings of a decrease in estimated clearance rates with age during the first few years of life in Tanzanian children [16], [19]. However durations decreased again in semi-immune adolescents and adults, though the oldest individuals had increased persistence. Overall, therefore there was no simple age-trend in duration, and the estimates of mean duration were all of the same order as the value of around 200 days for artificial *P. falciparum* infections in malariatherapy patients [37], [38]. This is also consistent with persistence of sub-patent asymptomatic infections in adults throughout the dry season in areas where transmission is restricted to only a few weeks per year [39].

Overall, therefore, age patterns in both FOI and in duration are not very marked when the analysis allows for age dependence in detectability. The generally higher parasite densities in children provide the most obvious explanation of why detectability decreases gradually with age [40], although detectibility and density do not seem to have exactly the same age-dependence. We conclude therefore, that most of the age-dependence in the patterns of appearance and disappearance of parasite clones is driven by the age-pattern of asexual parasite densities.

On the basis of well established results from elsewhere, the age patterns of parasite densities are the outcome of naturally-acquired anti-blood stage immunity. By implication, the effects of acquired immunity on the rates of acquisition and clearance of parasite clones are not very pronounced. At first sight it is difficult to understand how acquisition of immunity could have so little effect on persistence, but this ignores the complex dynamics of single infections driven by antigenic variation [41]. The models used here significantly simplify these dynamics, for instance, by treating parasite clearance and detection as independent of age of the infection, equivalent to assuming an exponential distribution of durations. In fact, further analyses have found that Gompertz or Weibull distributions of the duration are more realistic than the exponential ones [21], [34] but provide similar estimates of average duration (while being more difficult to fit).

These age patterns challenge the ideas that transmission in endemic areas is controlled by acquired pre-erythrocytic immunity, or by rapid clearance of infections in semi-immune hosts. We contend that most of the effect of acquired immunity on transmission is also likely to mediated by effects on parasite densities. Immunity also has direct effects on sexual stages of the parasite [42] but since the gametocyte stages of the parasite arise by developmental switching of asexual parasites, reduction in asexual biomass leads to fewer gametocytes and hence less potential to transmit to mosquitoes. This can be seen clearly in the data from artificial infections of humans [43]. A consequence is that the infectiousness of the humans host in endemic areas depends on the age of the host, with adults transmitting less than children [44]. Reduction of asexual blood stage densities is very likely the main effect of immunity driving the difference between naive and highly exposed hosts in the extent to which *P. falciparum* infections are propagated.

Both vector control and chemotherapeutic interventions act on transmission by changing FOI and/or duration of infection. Studies of these quantities therefore underpin models of the natural history of *P. falciparum* that are needed for projecting the likely impact of novel interventions and scale-up of existing ones. Apart from the Navrongo studies, we have so far applied methods allowing for imperfect detectability only to data covering only limited age ranges from Tanzania. There is need to test generalisability to areas with different patterns of transmission, if possible, linking estimates of FOI and duration to better measures of immune status and exposure.

## Supporting Information

Table S1 **Genotypes detected.** List of the 103 msp2 genotypes detected in a cohort study in 347 individuals from northern Ghana, their fragment sizes, allelic frequencies and the number of observations for each genotype per survey round and in total.
(DOC)
