# Supplementary material for: The Dynamics of Natural Plasmodium falciparum Infections
Source: PLoS One. 2012 Sep 18;7(9):e45542. doi: 10.1371/journal.pone.0045542 (PMC3445515; doi:10.1371/journal.pone.0045542)
Supplement: Table S1 — Genotypes detected. List of the 103 msp2 genotypes detected in a cohort study in 347 individuals from northern Ghana, their fragment sizes, allelic frequencies and the number of observations for each genotype per survey round and in total. (DOC) [file pone.0045542.s001.doc]

**Table S1. msp2 genotypes detected**

| Name of Genotype | Allelic Family | Mean Observed Size | **Allelic Frequency %** | Occurrences in Round 1 | Occurrences in Round 2 | Occurrences in Round 3 | Occurrences in Round 4 | Occurrences in Round 5 | Occurences in Round 6 | Total occurences all Rounds |
| --- | --- | --- | --- | --- | --- | --- | --- | --- | --- | --- |
| Fc27genotype297.3 | Fc27 | 298.2 | 1.86 | 15 | 32 | 27 | 16 | 16 | 13 | 119 |
| Fc27genotype300.3 | Fc27 | 300.0 | 0.06 | 2 | 0 | 1 | 0 | 0 | 1 | 4 |
| Fc27genotype309.3 | Fc27 | 308.6 | 0.36 | 3 | 4 | 7 | 6 | 1 | 2 | 23 |
| Fc27genotype312.3 | Fc27 | 313.2 | 0.38 | 3 | 3 | 6 | 5 | 3 | 4 | 24 |
| Fc27genotype318.3 | Fc27 | 317.9 | 0.47 | 4 | 8 | 2 | 5 | 6 | 5 | 30 |
| Fc27genotype327.3 | Fc27 | 327.1 | 0.50 | 9 | 7 | 7 | 2 | 2 | 5 | 32 |
| Fc27genotype333.3 | Fc27 | 332.5 | 0.17 | 0 | 1 | 3 | 0 | 4 | 3 | 11 |
| Fc27genotype336.3 | Fc27 | 335.9 | 10.19 | 100 | 133 | 134 | 100 | 93 | 91 | 651 |
| Fc27genotype345.3 | Fc27 | 346.0 | 0.13 | 0 | 4 | 1 | 3 | 0 | 0 | 8 |
| Fc27genotype351.3 | Fc27 | 351.8 | 0.27 | 3 | 3 | 3 | 1 | 7 | 0 | 17 |
| Fc27genotype354.3 | Fc27 | 355.1 | 0.13 | 0 | 5 | 0 | 3 | 0 | 0 | 8 |
| Fc27genotype360.3 | Fc27 | 359.3 | 1.72 | 21 | 20 | 14 | 20 | 17 | 18 | 110 |
| Fc27genotype366.3 | Fc27 | 365.3 | 0.13 | 1 | 0 | 1 | 0 | 6 | 0 | 8 |
| Fc27genotype372.3 | Fc27 | 372.0 | 5.76 | 66 | 65 | 73 | 55 | 56 | 53 | 368 |
| Fc27genotype375.3 | Fc27 | 373.9 | 0.08 | 0 | 2 | 2 | 0 | 1 | 0 | 5 |
| Fc27genotype381.3 | Fc27 | 383.0 | 0.31 | 2 | 3 | 2 | 5 | 4 | 4 | 20 |
| Fc27genotype384.3 | Fc27 | 385.2 | 0.06 | 0 | 1 | 0 | 0 | 2 | 1 | 4 |
| Fc27genotype387.3 | Fc27 | 388.6 | 0.11 | 0 | 2 | 1 | 2 | 0 | 2 | 7 |
| Fc27genotype393.3 | Fc27 | 394.6 | 0.19 | 4 | 2 | 2 | 3 | 0 | 1 | 12 |
| Fc27genotype399.3 | Fc27 | 399.2 | 0.25 | 3 | 2 | 2 | 2 | 3 | 4 | 16 |
| Fc27genotype405.3 | Fc27 | 405.6 | 0.31 | 4 | 2 | 4 | 3 | 2 | 5 | 20 |
| Fc27genotype408.3 | Fc27 | 408.2 | 1.27 | 9 | 23 | 17 | 11 | 9 | 12 | 81 |
| Fc27genotype417.3 | Fc27 | 418.0 | 7.08 | 67 | 97 | 85 | 80 | 53 | 70 | 452 |
| Fc27genotype423.3 | Fc27 | 424.5 | 0.05 | 0 | 0 | 1 | 1 | 0 | 1 | 3 |
| Fc27genotype447.3 | Fc27 | 448.4 | 0.06 | 0 | 1 | 2 | 0 | 1 | 0 | 4 |
| Fc27genotype456.3 | Fc27 | 455.1 | 0.33 | 3 | 6 | 4 | 4 | 1 | 3 | 21 |
| Fc27genotype462.3 | Fc27 | 461.3 | 0.06 | 0 | 0 | 3 | 0 | 0 | 1 | 4 |
| Fc27genotype516.3 | Fc27 | 517.3 | 0.33 | 1 | 6 | 7 | 3 | 2 | 2 | 21 |
| 3D7genotype206 | 3D7 | 205.5 | 0.66 | 5 | 9 | 4 | 11 | 6 | 7 | 42 |
| 3D7genotype209 | 3D7 | 208.1 | 0.20 | 3 | 2 | 2 | 2 | 2 | 2 | 13 |
| 3D7genotype212 | 3D7 | 211.7 | 0.60 | 7 | 7 | 9 | 5 | 6 | 4 | 38 |
| 3D7genotype215 | 3D7 | 215.0 | 0.16 | 3 | 3 | 1 | 1 | 1 | 1 | 10 |
| 3D7genotype218 | 3D7 | 217.4 | 0.74 | 8 | 8 | 7 | 11 | 4 | 9 | 47 |
| 3D7genotype221 | 3D7 | 220.0 | 0.17 | 1 | 4 | 1 | 2 | 2 | 1 | 11 |
| 3D7genotype224 | 3D7 | 222.9 | 0.86 | 12 | 9 | 9 | 10 | 6 | 9 | 55 |
| 3D7genotype227 | 3D7 | 226.5 | 0.44 | 14 | 3 | 3 | 4 | 3 | 1 | 28 |
| 3D7genotype230 | 3D7 | 228.8 | 0.86 | 12 | 14 | 2 | 10 | 7 | 10 | 55 |
| 3D7genotype233 | 3D7 | 232.7 | 0.39 | 7 | 3 | 1 | 7 | 4 | 3 | 25 |
| 3D7genotype236 | 3D7 | 235.2 | 2.63 | 29 | 30 | 31 | 26 | 25 | 27 | 168 |
| 3D7genotype239 | 3D7 | 239.0 | 0.38 | 1 | 6 | 3 | 5 | 6 | 3 | 24 |
| 3D7genotype242 | 3D7 | 241.1 | 1.25 | 9 | 19 | 17 | 16 | 9 | 10 | 80 |
| 3D7genotype245 | 3D7 | 244.6 | 2.41 | 29 | 26 | 30 | 31 | 22 | 16 | 154 |
| 3D7genotype248 | 3D7 | 247.4 | 1.33 | 21 | 23 | 12 | 14 | 10 | 5 | 85 |
| 3D7genotype251 | 3D7 | 250.8 | 0.91 | 12 | 11 | 7 | 15 | 7 | 6 | 58 |
| 3D7genotype254 | 3D7 | 253.1 | 2.00 | 22 | 30 | 24 | 19 | 18 | 15 | 128 |
| 3D7genotype257 | 3D7 | 257.8 | 1.88 | 22 | 19 | 22 | 24 | 18 | 15 | 120 |
| 3D7genotype260 | 3D7 | 260.1 | 0.39 | 6 | 4 | 4 | 6 | 4 | 1 | 25 |
| 3D7genotype263 | 3D7 | 262.5 | 3.57 | 33 | 47 | 55 | 39 | 27 | 27 | 228 |
| 3D7genotype266 | 3D7 | 265.4 | 1.86 | 16 | 22 | 18 | 27 | 19 | 17 | 119 |
| 3D7genotype269 | 3D7 | 269.0 | 1.46 | 18 | 20 | 19 | 16 | 11 | 9 | 93 |
| 3D7genotype272 | 3D7 | 271.3 | 3.62 | 49 | 42 | 39 | 44 | 23 | 34 | 231 |
| 3D7genotype275 | 3D7 | 276.4 | 3.27 | 34 | 43 | 44 | 38 | 20 | 30 | 209 |
| 3D7genotype278 | 3D7 | 280.0 | 3.05 | 38 | 49 | 31 | 37 | 20 | 20 | 195 |
| 3D7genotype281 | 3D7 | 281.6 | 1.08 | 14 | 19 | 13 | 12 | 5 | 6 | 69 |
| 3D7genotype284 | 3D7 | 284.4 | 0.77 | 13 | 8 | 8 | 9 | 7 | 4 | 49 |
| 3D7genotype287 | 3D7 | 287.3 | 1.64 | 18 | 25 | 23 | 13 | 12 | 14 | 105 |
| 3D7genotype290 | 3D7 | 290.1 | 1.89 | 26 | 27 | 22 | 21 | 14 | 11 | 121 |
| 3D7genotype293 | 3D7 | 293.4 | 1.25 | 12 | 13 | 12 | 19 | 11 | 13 | 80 |
| 3D7genotype296 | 3D7 | 296.3 | 1.61 | 19 | 20 | 19 | 20 | 13 | 12 | 103 |
| 3D7genotype299 | 3D7 | 299.4 | 1.91 | 17 | 26 | 21 | 26 | 15 | 17 | 122 |
| 3D7genotype302 | 3D7 | 302.4 | 0.75 | 9 | 9 | 13 | 6 | 4 | 7 | 48 |
| 3D7genotype305 | 3D7 | 305.6 | 1.61 | 19 | 22 | 19 | 17 | 11 | 15 | 103 |
| 3D7genotype308 | 3D7 | 308.4 | 0.89 | 10 | 11 | 10 | 8 | 6 | 12 | 57 |
| 3D7genotype311 | 3D7 | 311.4 | 1.25 | 14 | 17 | 16 | 15 | 7 | 11 | 80 |
| 3D7genotype314 | 3D7 | 314.6 | 0.53 | 8 | 4 | 6 | 8 | 2 | 6 | 34 |
| 3D7genotype317 | 3D7 | 317.2 | 1.22 | 13 | 13 | 22 | 13 | 9 | 8 | 78 |
| 3D7genotype320 | 3D7 | 320.8 | 0.47 | 6 | 7 | 7 | 9 | 1 | 0 | 30 |
| 3D7genotype323 | 3D7 | 323.3 | 0.92 | 7 | 13 | 7 | 13 | 10 | 9 | 59 |
| 3D7genotype326 | 3D7 | 326.5 | 0.70 | 4 | 7 | 13 | 12 | 6 | 3 | 45 |
| 3D7genotype329 | 3D7 | 329.6 | 0.83 | 7 | 12 | 6 | 10 | 10 | 8 | 53 |
| 3D7genotype332 | 3D7 | 332.7 | 0.53 | 7 | 5 | 3 | 5 | 8 | 6 | 34 |
| 3D7genotype335 | 3D7 | 335.6 | 0.85 | 14 | 8 | 10 | 7 | 5 | 10 | 54 |
| 3D7genotype338 | 3D7 | 338.2 | 0.27 | 2 | 8 | 2 | 4 | 0 | 1 | 17 |
| 3D7genotype341 | 3D7 | 341.8 | 0.80 | 11 | 10 | 11 | 10 | 5 | 4 | 51 |
| 3D7genotype344 | 3D7 | 344.7 | 0.33 | 6 | 4 | 7 | 2 | 1 | 1 | 21 |
| 3D7genotype347 | 3D7 | 347.2 | 1.00 | 9 | 11 | 15 | 8 | 7 | 14 | 64 |
| 3D7genotype350 | 3D7 | 351.3 | 0.38 | 3 | 9 | 4 | 3 | 3 | 2 | 24 |
| 3D7genotype353 | 3D7 | 353.4 | 0.60 | 7 | 11 | 6 | 6 | 2 | 6 | 38 |
| 3D7genotype356 | 3D7 | 356.5 | 0.38 | 3 | 4 | 6 | 3 | 4 | 4 | 24 |
| 3D7genotype359 | 3D7 | 359.8 | 0.44 | 7 | 5 | 7 | 3 | 2 | 4 | 28 |
| 3D7genotype362 | 3D7 | 362.2 | 0.31 | 2 | 4 | 6 | 3 | 1 | 4 | 20 |
| 3D7genotype365 | 3D7 | 364.7 | 0.91 | 4 | 12 | 14 | 12 | 7 | 9 | 58 |
| 3D7genotype368 | 3D7 | 368.2 | 0.30 | 1 | 7 | 5 | 2 | 1 | 3 | 19 |
| 3D7genotype371 | 3D7 | 371.3 | 0.42 | 5 | 6 | 5 | 5 | 3 | 3 | 27 |
| 3D7genotype377 | 3D7 | 375.6 | 0.85 | 10 | 12 | 9 | 12 | 5 | 6 | 54 |
| 3D7genotype380 | 3D7 | 381.4 | 0.94 | 11 | 14 | 11 | 10 | 5 | 9 | 60 |
| 3D7genotype383 | 3D7 | 383.9 | 0.20 | 1 | 1 | 6 | 2 | 2 | 1 | 13 |
| 3D7genotype386 | 3D7 | 386.9 | 0.39 | 5 | 8 | 6 | 2 | 2 | 2 | 25 |
| 3D7genotype389 | 3D7 | 389.3 | 0.23 | 3 | 4 | 3 | 2 | 1 | 2 | 15 |
| 3D7genotype392 | 3D7 | 392.7 | 0.33 | 5 | 5 | 3 | 2 | 2 | 4 | 21 |
| 3D7genotype395 | 3D7 | 395.5 | 0.27 | 1 | 4 | 7 | 3 | 2 | 0 | 17 |
| 3D7genotype398 | 3D7 | 398.5 | 0.53 | 2 | 8 | 8 | 8 | 3 | 5 | 34 |
| 3D7genotype404 | 3D7 | 404.0 | 0.16 | 2 | 1 | 3 | 1 | 2 | 1 | 10 |
| 3D7genotype407 | 3D7 | 407.1 | 0.27 | 2 | 10 | 1 | 2 | 1 | 1 | 17 |
| 3D7genotype410 | 3D7 | 410.1 | 0.17 | 0 | 3 | 3 | 0 | 2 | 3 | 11 |
| 3D7genotype413 | 3D7 | 412.9 | 0.11 | 0 | 1 | 2 | 1 | 1 | 2 | 7 |
| 3D7genotype416 | 3D7 | 416.1 | 0.30 | 3 | 5 | 3 | 4 | 1 | 3 | 19 |
| 3D7genotype422 | 3D7 | 421.9 | 0.19 | 1 | 2 | 3 | 1 | 3 | 2 | 12 |
| 3D7genotype428 | 3D7 | 428.3 | 0.16 | 1 | 5 | 0 | 2 | 0 | 2 | 10 |
| 3D7genotype431 | 3D7 | 430.9 | 0.08 | 2 | 1 | 2 | 0 | 0 | 0 | 5 |
| 3D7genotype434 | 3D7 | 434.4 | 0.13 | 0 | 2 | 2 | 2 | 0 | 2 | 8 |
| 3D7genotype437 | 3D7 | 436.4 | 0.05 | 0 | 2 | 0 | 0 | 1 | 0 | 3 |
| 3D7genotype446 | 3D7 | 446.1 | 0.09 | 1 | 2 | 1 | 1 | 0 | 1 | 6 |
